# The agents of Sever Acute Respiratory Infection cases in Ben Tre province 2008-2010

**Published:** 2011-01-10

**Authors:** Tran Ngoc Huu, Vu Thi Que Huong, Do Kien Quoc, Phan Van Tinh, Nguyen Thanh Long, Nguyen Thi Kim Hoang, Luong Chan Quang

**Affiliations:** 1 Pasteur Institute of Ho Chi Minh City, Vietnam

## 

Since 2003, Southern Vietnam has become the most serious affected area by various emerging diseases such as: A/H5N1, SARS, Rubella, etc. Therefore, the establishment of emerging disease surveillance is essential and necessary to provide scientific information for good understand on the emerging diseases and for effective outbreak response.

A specific surveillance system for Severe Acute Respiratory Infection (SARI) was established in 02 sentinel hospitals in Ben Tre province from August 2008 to June 2010. Patient’s signature on written ICF had been required before the enrollment. All of the enrolled SARI cases were investigated and their throat swabs were collected for testing. Multiplex RT-PCR was applied to determine 17 viral agents. In addition, blood culture has been performed since August 2009 in order to detect bacterial agents on these SARI cases.

Out of 1,842 SARI cases enrolled into the surveillance system, 969 (52.61%) cases had positive testing results, including: 913 (49.75%) cases had positive testing results with viral agents and 56 (9.89%) of 566 blood culture specimens had positive testing results with bacterial agents. Moreover, the proportion of co-infection between virus and bacteria was 0.98%. There were 14 viral agents had been detected including: Inf A/H1N1pdm (1.41%), Inf A (2.61%), Inf B (0.98%), Inf C (0.11%), hRV (18.1%), RSV (14.13%), Boca (3.32%), Adeno (1.68%), Entero (0.65%), Corona HKUI (0.11%), hMPV (2.28%), Parainfluenza type 1 (1.58%), Parainfluenza type 3 (2.23%) and Parainfluenza type 4 (0.33%) viruses. A remarkable attention is that hRV and RSV which were identified as the cause of some sudden deaths in infants in China, Philippines and respiratory outbreak in Cambodia in 2008 were 02 predominant agents.

The major concern is that Inf A virus in the South predominates in summer season and this is very different from other regions in Vietnam and even other parts of the world. In addition, viral agents were shifted by seasons: Boca virus (January to April), Inf A (May to July) and RSV (August to November). The results of the surveillance show that the viral pathogens of SARI are variety and most of them have never been detected in the South of Vietnam. In addition, all types of Influenza virus are detected on SARI cases and the viral pathogens vary by the seasons.

